# Confinement Effects on Chemical Equilibria: Pentacyano(Pyrazine)Ferrate(II) Stability Changes within Nanosized Droplets of Water

**DOI:** 10.3390/molecules23040858

**Published:** 2018-04-09

**Authors:** Teofilo Borunda, Alexander J. Myers, J. Mary Fisher, Debbie C. Crans, Michael D. Johnson

**Affiliations:** 1 Department of Chemistry & Biochemistry, New Mexico State University, Las Cruces, NM 88003, USA; teo2491.tb@gmail.com (T.B.); amwy6@mail.missouri.edu (A.J.M.); 2 Department of Chemistry, Colorado State University, Ft. Collins, CO 80523, USA; maryjfisher@gmail.com

**Keywords:** Confinement effects, coordination chemistry at the nanoscale, pentacyano(pyrazine)ferrate(II), [Fe(CN)_5_pyz]^3−^, hydrolysis rates, monomer-dimer equilibrium, reverse micelles, interface interactions

## Abstract

Nanoscale confinement is known to impact properties of molecules and we observed changes in the reactivity of an iron coordination complex, pentacyano(pyrazine)ferrate(II). The confinement of two coordination complexes in a sodium AOT/isooctane reverse micellar (RM) water droplet was found to dramatically increase the hydrolysis rate of [Fe(CN)_5_pyz]^3−^ and change the monomer-dimer equilibria between [Fe(CN)_5_pyz]^3−^ and [Fe_2_(CN)_10_pyz]^6−^. Combined UV-Vis and ^1^H-NMR spectra of these complexes in RMs were analyzed and the position of the monomer-dimer equilibrium and the relative reaction times were determined at three different RM sizes. The data show that the hydrolysis rates (loss of pyrazine) are dramatically enhanced in RMs over bulk water and increase as the size of the RM decreases. Likewise, the monomer-dimer equilibrium changes to favor the formation of dimer as the RM size decreases. We conclude that the effects of the [Fe(CN)_5_pyz]^3−^ stability is related to its solvation within the RM.

## 1. Introduction

Coordination complexes undergo ligand exchange reactions that are critical for their chemical reactivity in catalytic processes [1,2,3,4]. Mechanistic studies of metal–ligand complexation reactions have been reported for bulk solvent systems, water in particular, and are the basis for our understanding of how metal complexes behave in solution [5,6,7,8,9,10,11,12]. While such studies have provided an understanding of the fundamental ligand exchange reaction mechanisms, and hence metal ion speciation and behavior, they may not capture all the effects present in systems where inhomogeneous bulk solvation of metal ions governs the system properties. This may be particularly true for example at enzyme active sites, and at interfaces where chemical reactivity may be different from that found in bulk solvent media. Conditions where reaction solvation has been altered by “confinement” generally results in changes in chemical reactivity [13,14,15,16,17]. Fendler et al. reported accelerated substitution aquation rates for chromium(III) and cobalt(III) amine complexes in reverse micelles (RM) [18,19]. The increased substitution rates were attributed to association of the coordinated ligand with the surfactant RM interfaces and solvation changes. In other reports, small accelerations in ligand substitution rates at pentacyano(ligand)ferrate(II) centers were reported [20]. The substitution reactions of labile cobalt(II) complexes were also determined [21,22] and the observed differences in their substitution rates and speciation within RMs were ascribed to the cobalt complex residing at the interface where its solvation is different than in bulk solvents. This may also hold true for the iron pentacyano(ligand)ferrate(II) studies described above. We have previously found that compounds can either be more stable [23,24] or hydrolyze [25] in the vicinity of a reverse micellar interface. It is important to investigate such systems to understand the underlying factors affecting the reactions of complexes in nanoenvironment.

We have initiated a series of studies of how chemical reactivity of transition metal complexes is affected when placed into RMs, where the probe of interest is confined within a nano-sized water pool or near its interface [26,27,28]. These studies have shown both rate enhancement and diminution, depending on the system under investigation. The rate changes have been ascribed due to differences in the location of the probe molecules (complexes and free ligands) as well as changes in their fundamental physical properties, i.e., pKa values [29] or redox potentials [30]. As a result of these studies, it has become clear that more detailed information on simple substitution reactions at metal centers in coordination complexes is essential to understand the molecular reactivity modifications we have observed.

**Scheme 1:**
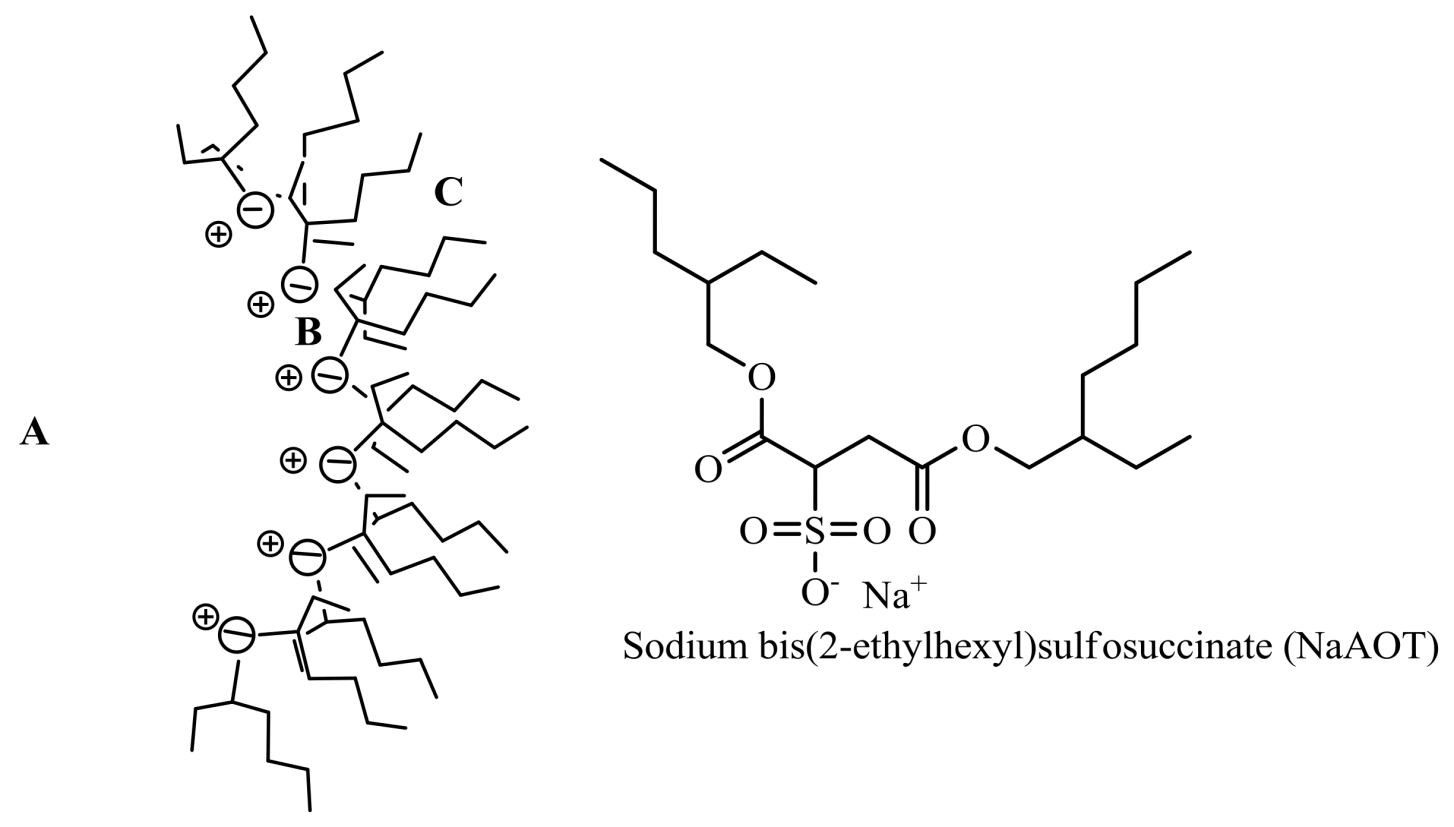
The structures of the reverse micellar interface of a NaAOT reverse micelle (RM) with three different specific locations for additives. Labels A, B, and C shown are in the aqueous core, interfacial region, and hydrophobic part of the nanostructure, respectively. (**left**) and the structure of NaAOT (**right**).

RMs are self-assembled structures formed when small amounts of water are added to a solution of a surfactant in an organic solvent. A large number of such systems can be formed, the most studied and best characterized system consist of sodium bis(ethylhexyl)sulfosuccinae (NaAOT), organic hydrocarbon solvent, and water [31,32,33,34,35,36,37,38]. Combining these three components, the surfactant rapidly self-assembles to surround the water droplets as RMs suspended in the organic solvent. The structure of NaAOT and a representative “slice” of the structure of a NaAOT RM interface is shown in Scheme 1. Although the image represents a seemingly rigid spherical structure, the system is thermodynamically stable but water and potential probes and surfactants are exchanging rapidly between RMs. Importantly, droplet size, and hence water content, is easily changed in a controlled manner. Specifically, the water droplet size is directly proportional to the ratio of water to surfactant (*w*_0_ = [H_2_O]/[surfactant]) [29,32,39].

Coordination complexes of transition metal ions can be particularly sensitive to their environment, so their confinement within nanosized water droplet cores of RMs allows a change in speciation [40,41]. Since ligand exchange will be sensitive to environment, confinement could change the complexation equilibria as a result of interaction with the interface, smaller water pool, or both [42]. Characterization of systems that may undergo speciation changes are thus desirable in order to examine if their changes in molecular properties are associated with information on the location of the compounds in these RM systems. In the following, we report the partial loss of pyrazine and subsequent dimerization of pentacyano(pyrazine)ferrate(II), as shown below in Equation (1), along with the effect of the size of the RM on the position of this equilibrium. These studies are important because they demonstrate how these nanosized nonhomogeneous environments can change the equilibria in coordination complexes important in catalytic reactions.

2 [Fe(CN)_5_(pyrazine)]^3−^ ⇌ [Fe(CN)_5_(pyrazine)Fe(CN)_5_]^6−^ + pyrazine (1)

(M = monomer)  (D = dimer)    (Pyz)

## 2. Results and Discussion

Aqueous solutions of sodium pentacyano(pyrazine)ferrate(II) are relatively stable and decompose slowly (k_d_ = 4.2 × 10^−4^ s^−1^) with the release of pyrazine as shown in Equation (2) [43].

[Fe(CN)_5_pyz]^3−^ ⇌ [Fe(CN)_5_OH_2_]^3−^ + pyz (2)

As noted, the products and reactants are in equilibrium with each other (K_f_ = 90) with a reformation rate constant of k_f_ = 3.8 × 10^2^ M^−1^ s^−1^ [43]. The well-known aquation rates, coupled with a high molar extinction coefficient of the MLCT band at 458 nm, 3 × 10^3^ M^−1^ cm^−1^ [20], make [Fe(CN)_5_pyz]^3−^ an excellent system to study the effects of confinement on the stability of coordination complexes. In addition, the pK_a_ of free pyrazine is 0.6, and the pK_a_ of complexed pyrazine is even lower, making this equilibrium relatively pH insensitive.

### 2.1. Kinetic Measurements Using UV-Vis Spectroscopy

**Figure 1:**
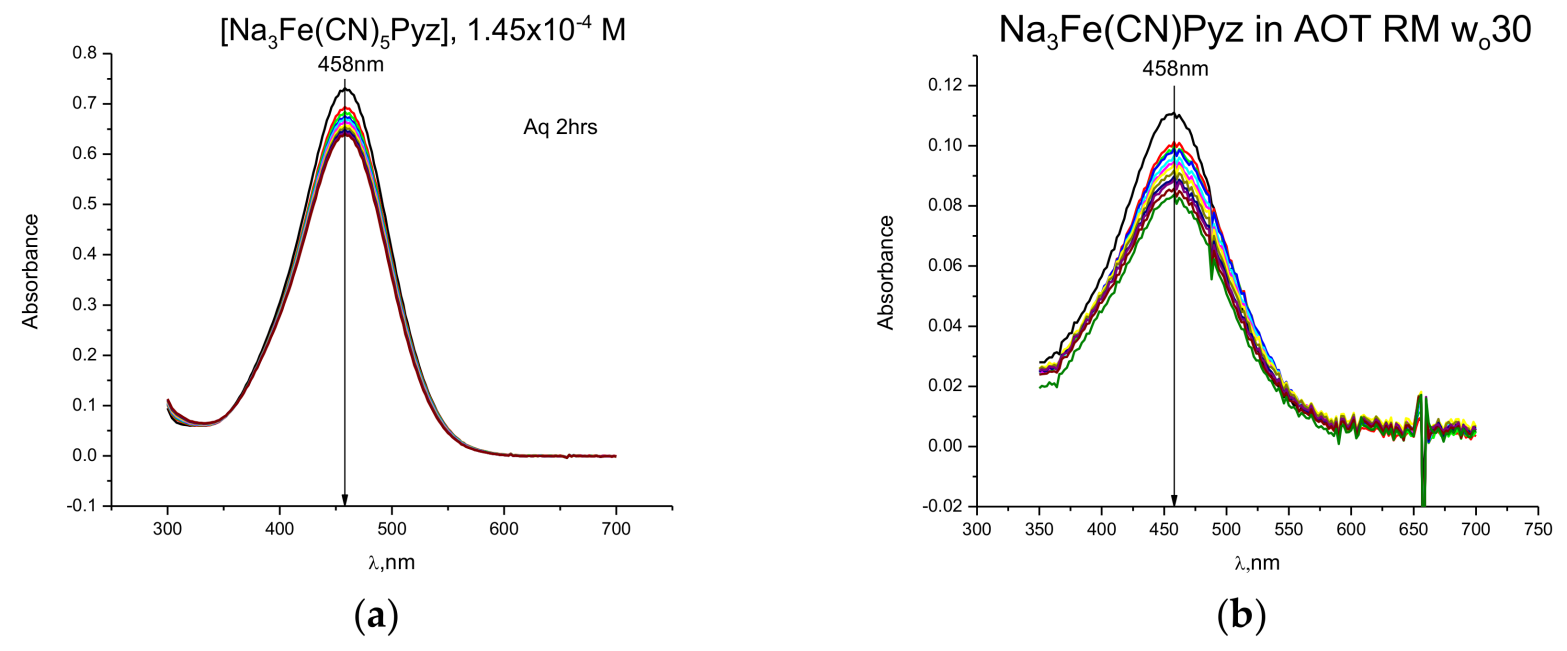
Hydrolysis of aqueous (**a**) and confined solutions (**b**) of an iron-pyrazine complex in 200 mM AOT in isooctane. The concentration of Na_3_[Fe(CN)_5_pyz] is 2.45 × 10^−4^ M and approximately 5 × 10^−4^ M in the AOT/isooctane RM suspension.

When Na_3_[Fe(CN)_5_pyz] is dissolved in water at ~5 × 10^−4^ M concentration, the MLCT peak at 458 nm slowly disappears as the pyrazine is lost from the first coordination sphere, see Figure 1. The extent of hydrolysis of the metal complex in water and upon confinement within a large AOT RM can readily be monitored using UV-Vis in the absence and the in presence of compound. Both sets of spectra were recorded over a 2-h timeframe and shown in Figure 1.

In a large RM, *w*_0_ 30, the hydrolysis rate is very similar to bulk water, see Figure 1. It is important to note that the position of the peak remains at 458 nm which suggests that the pyrazine portion of the complex has not penetrated the negatively charged interfacial region of the AOT-RM matrix. The extinction coefficients (calculated as probe molarity within the entire sample, not just the RM pool) in and out of a RM are identical, again supporting the location of the probe within the water pool. It is well known that MLCT complexes such as these are very sensitive to their environment and that penetration/insertion into the interface should result in a change of the MLCT band [11,20]. This is, in fact, what is observed with other MLCT complexes in a range of different RMs [20].

**Figure 2:**
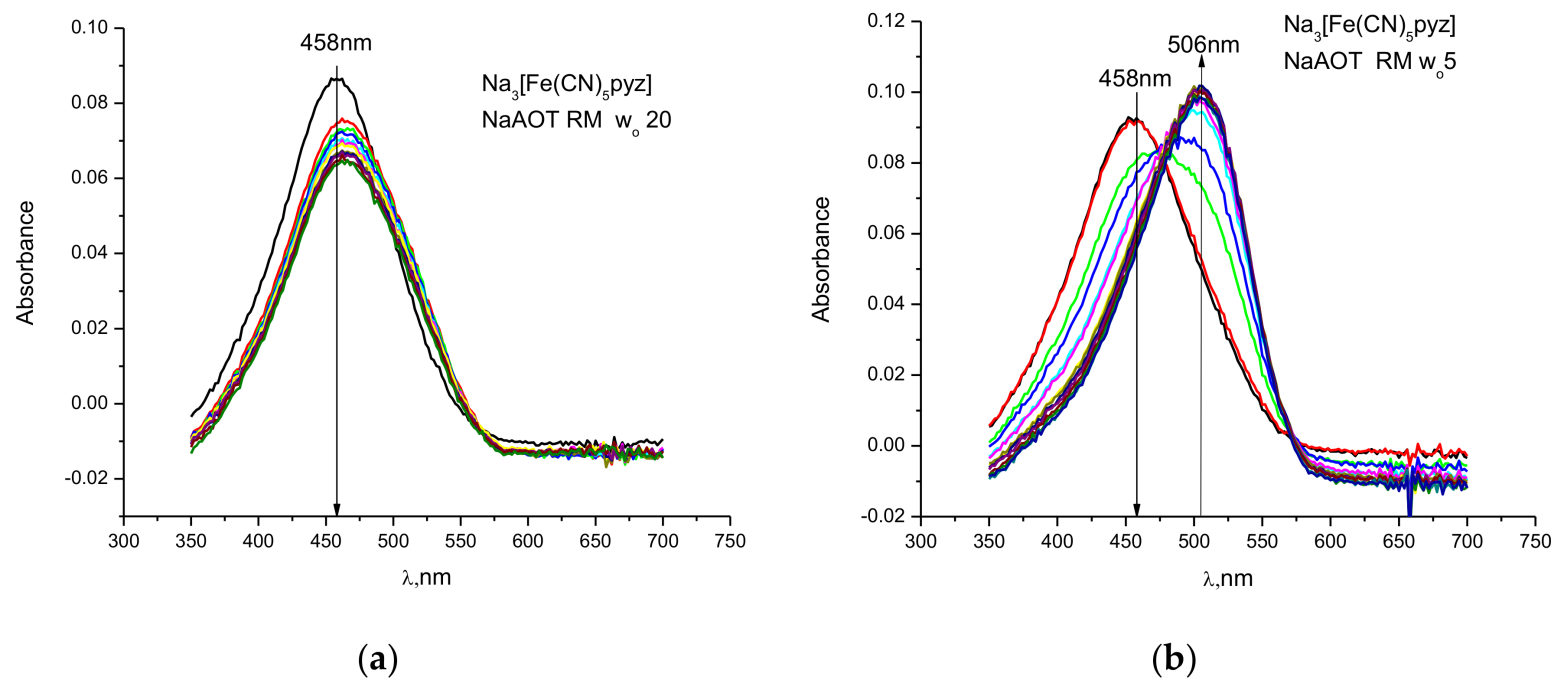
Hydrolysis of [Fe(CN)_5_pyz]^3−^ in *w*_0_ 20 (**a**) and in *w*_0_ 5 (**b**) AOT/isooctane. The water concentration of [Fe(CN)_5_pyz]^3−^ is approximately 10^−4^ M.

When the probe molecule is confined within a smaller RM, differences are immediately observed. Figure 2 shows the hydrolysis of the iron-pyrazine complex in two smaller AOT RMs with *w*_0_ sizes 20 and 5. At *w*_0_ 20, the hydrolysis occurs more extensively than in bulk water, over a two-hour time frame, and with the slight appearance of a red-shifted shoulder. When the size of the RM is small, *w*_0_ 5, the hydrolysis is complete within two hours and the shoulder observed at *w*_0_ 20 is now resolved into a new peak at 506 nm. The observed peak position corresponds to the literature value for the dimer, [Fe_2_(CN)_10_(pyz)]^6−^ [44]. 

The observed spectra may be explained by the initial hydrolysis of some of the starting material into pyrazine and the pentacyanoaquaferrate(II) complex. This aqua intermediate then rapidly adds to the remaining starting material to form the pyrazine bridged dimer.

H_2_O + [Fe(CN)_5_pyz]^3−^ ⇌ [Fe(CN)_5_OH_2_]^3−^ + pyz (3)

[Fe(CN)_5_OH_2_]^3−^ + [Fe(CN)_5_pyz]^3−^ ⇌ [Fe_2_(CN)_10_pyz]^6−^ + H_2_O (4)

The formation of the pyrazine dimer in simple aqueous solutions is readily measured. However, in RMs the conversion is more complex and the kinetic measurements are difficult to make. We suggest the complexities are due to a potential loss of cyanide from the iron center [11] or the formation of the oxo(hydroxo)bridged iron dimer, [(CN)_5_Fe-O(H)-Fe(CN)_5_]^(7−)/8−^ initially formed from the aqua species [11].

**Figure 3:**
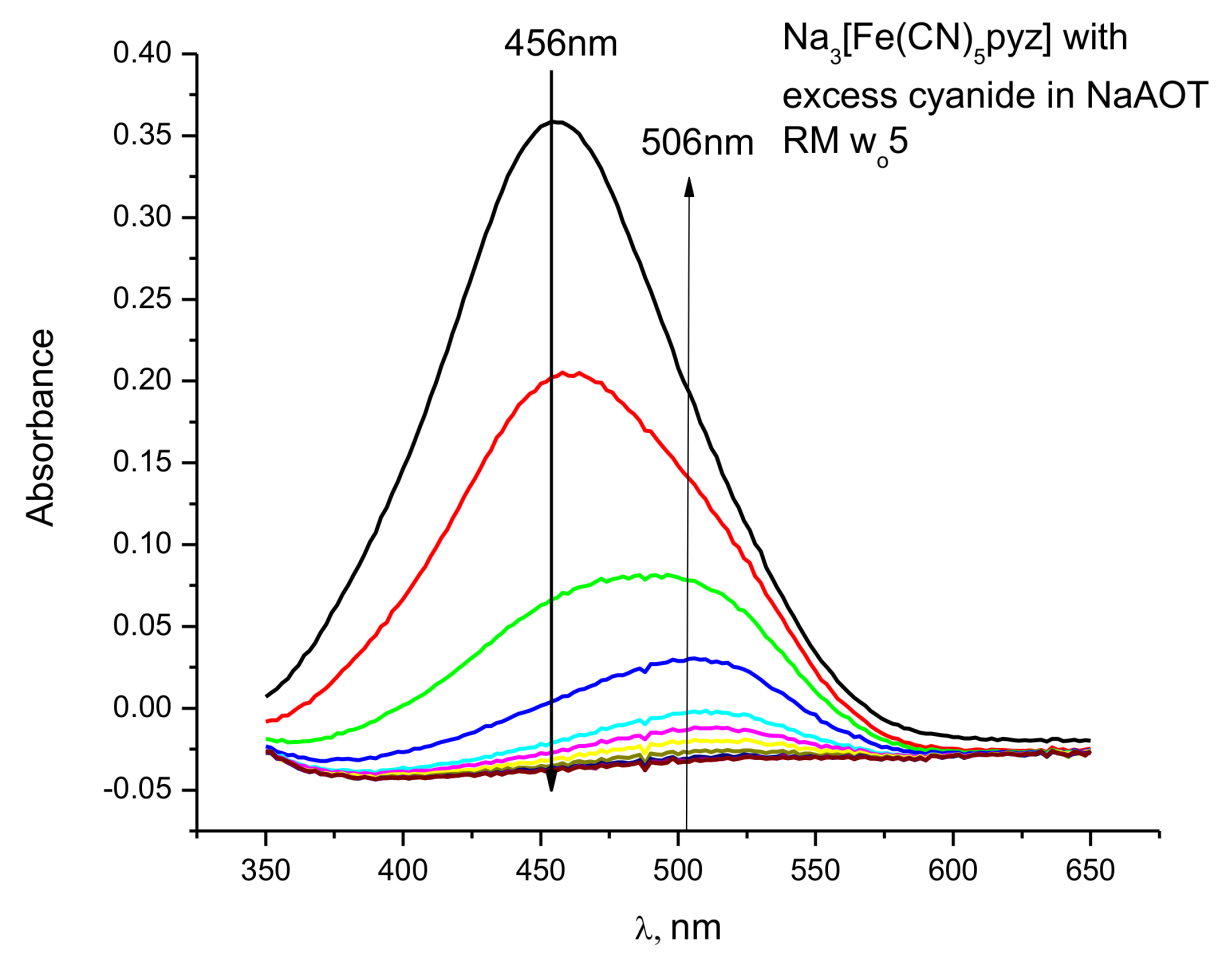
Trapping experiments for [Fe(CN)_5_OH_2_]^3−^ with 0.05M sodium cyanide in the water droplet in a *w*_0_ 5 AOT/isooctane RM. The concentration of [Fe(CN)_5_pyz]^3−^ is approximately 5 × 10^−5^ M.

Attempts to suppress the dimerization process were carried out by trapping the aqua intermediate with sodium cyanide, thereby converting it into the substitution inert hexacyanoferrate(II). In aqueous media with 0.050 M cyanide, the simple loss of the peak at 458 nm was observed. Figure 3 shows the same reaction carried out in a *w*_0_ 5 RM. Even under these conditions, dimer formation occurs, albeit to a much lesser extent. This observation supports the interpretation that the pyrazine bridged dimer is thermodynamically favored in the small RMs.

**Figure 4:**
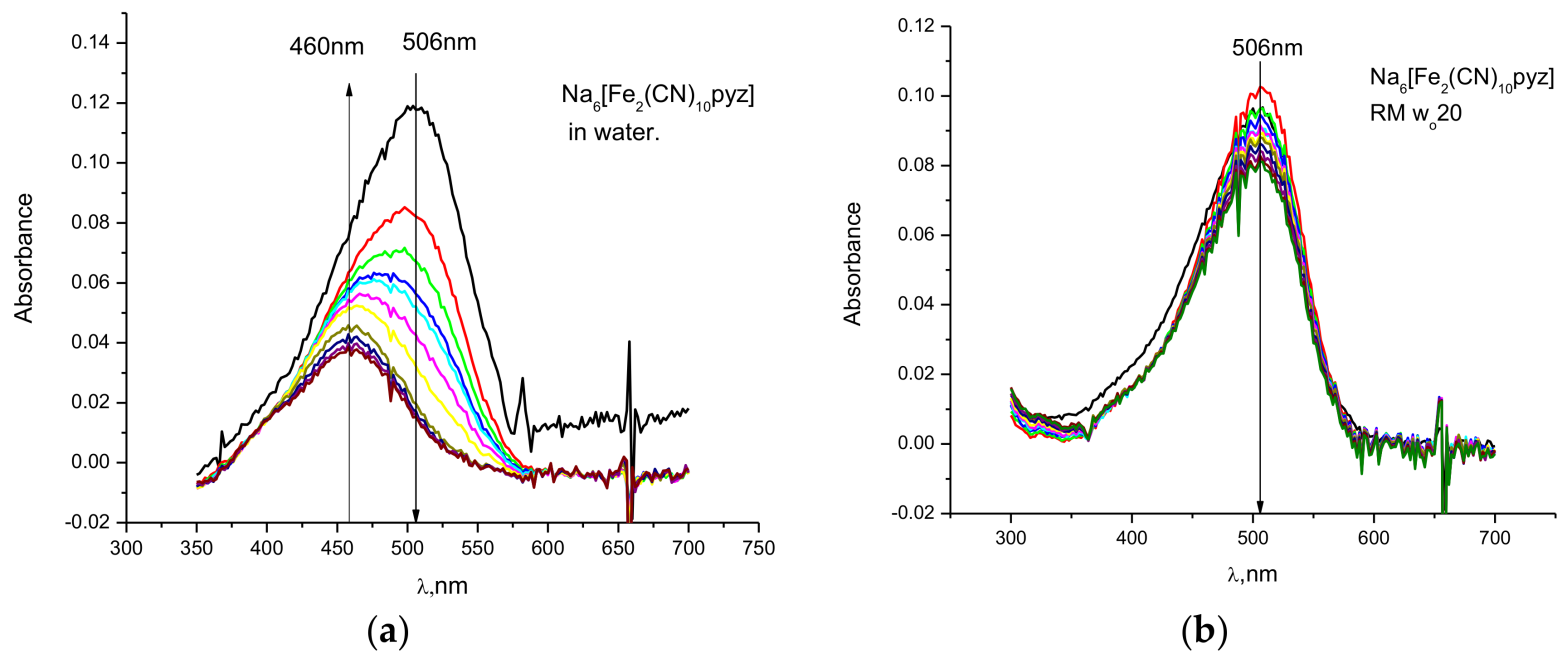
Hydrolysis of the ~5 × 10^−5^ M iron-pyrazine dimer in water (**a**) and a *w*_0_ 20 AOT/isooctane RM (**b**). The reaction was monitored for about 2 h.

Finally, starting with the pyrazine bridged dimer and dissolving it into water, the peak at 506 nm decreased and was replaced by a peak at 458 nm, corresponding to formation of the monomer. This is in stark contrast to observations with the dimer confined within a *w*_0_ 20 RM, see Figure 4. During the same time-period while the dimer hydrolyzed in bulk water, the dimer appeared to be more stable with only observation of about ~20% decomposition. These results provide evidence that the dimer’s stability is greatly enhanced upon confinement within an AOT RM. Additional information on where the complexes are located is desirable upon placement into the RMs.

### 2.2. Thermodynamic Measurements using ^1^H-NMR Spectroscopy

**Figure 5:**
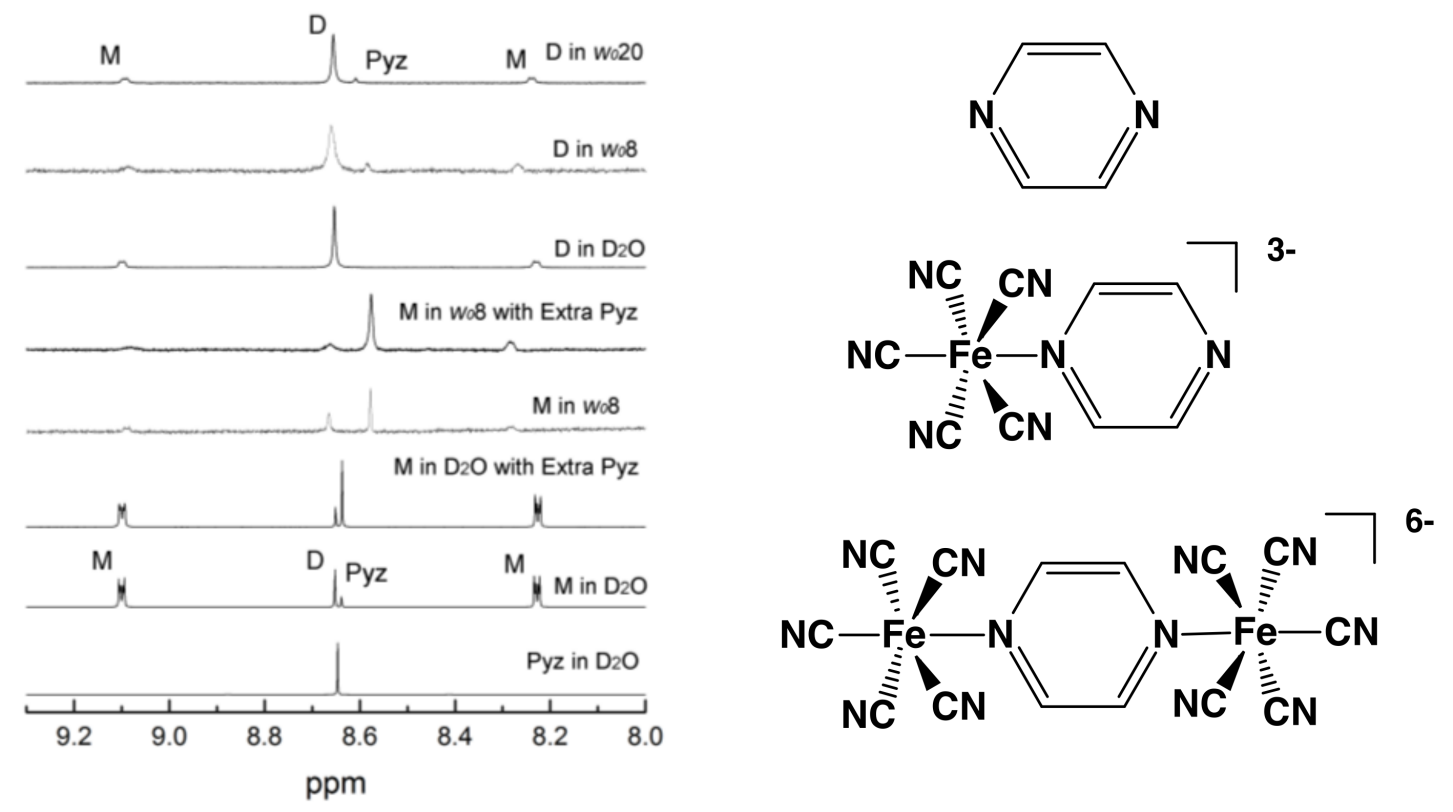
^1^H-NMR spectra recorded at 300 MHz of 20 mM M (or D) in aqueous solution, or in 0.75 M AOT /isooctane RMs. Starting from the bottom: Pyz in D_2_O, M in D_2_O, M in D_2_O spiked with Pyz; RM size *w*_0_ 8 and RM spiked with Pyz; D in D_2_O; D in RM *w*_0_ 8 and *w*_0_ 20. The structures of pyrazine (abbreviated Pyr), the monomeric pentacyano(pyrazine)ferrate(II) [Fe(CN)_5_]^3−^ (abbreviated M) and the dimeric [Fe(CN)_5_(pyrazine)Fe(CN)_5_]^6−^ (abbreviated D) are shown.

To gain further insight into these reactions in nanodroplets, we used ^1^H-NMR spectroscopy to determine the location of the components and the equilibrium constants. In Figure 5, the ^1^H-NMR spectra are shown for the iron monomer (abbreviated M on the figures), dimer (abbreviated D on the figures), and free pyrazine, (abbreviated Pyz on the figures) in D_2_O and a large and small RM, respectively. In Figure 5, the bottom three spectra provide for the assignment of the resonances of each species in D_2_O (M, D, and Pyz). The monomer shows the expected doublets at 9.09 and 8.22 ppm from the coordinated pyrazine, along with two other resonances at 8.65 and 8.63 ppm [45]. The signal at 8.63 ppm is assigned to free pyrazine and the dimer shows a singlet at 8.65 ppm. This assignment is further confirmed from spiking experiments where free pyrazine is added (1.4:1.0 for M:Pyz) and the resonance at 8.63 ppm increases. Within the time required to prepare the solutions and carry out the NMR experiment, it is clear that the monomer has partially aquated to form dimer and free pyrazine. In an aqueous solution dissolved monomer is about 75% and the dimer formed is 25% of the total iron speciation at the same concentration the experiments were done in RMs after about one to two hours as determined from the ratios of the integrations in the ^1^H-NMR spectra. 

We found that placing the monomer into an AOT/isooctane RMs rapidly decreases the monomer signal in the spectra with a concomitant increase in the dimer and free pyrazine signals. Because the peak positions shifted from those found in D_2_O, spiking experiments with pyrazine were again carried out to identify the free pyrazine peak. In a *w*_0_ 8 RM the resonances of the monomer and dimer were shifted only slightly. The monomer is now at 9.02 and 8.27 ppm and the dimer is at 8.66 ppm. These small changes (~0.06 ppm) support that there is little direct interaction with the AOT interface, as suggested by the lack of change of the MLCT band in the visible spectrum. Likewise, the free pyrazine shifts from 8.63 to 8.58 ppm, which also indicates it remains in the water pool of the RM. In experiments with aniline in RMs, the resonances shift as much as 0.3 ppm between bulk water and in *w*_0_ 10 RMs [29]. Similar shifts would be expected for the free and the monomer pyrazine resonances between D_2_O and RM *w*_0_ 8 if the Pyz was associated with the interface. Similar observations would be made if smaller ionic shells encapsulate ionic pools which provide smaller organized water pools that rigidly stabilized the iron complexes.

Similarly, the top three spectra in Figure 5 show the dimer in D_2_O, the dimer in an AOT/isooctane RM, and the dimer in an AOT/isooctane RM with extra Pyz added. These spectra demonstrate the chemical shifts for the dimer in D_2_O and a *w*_0_ 8 RM are essentially identical. This suggests that the dimer is in the water pool with little interaction between the interface and the pyrazine bridge. In both *w*_0_ 8 and 20 RMs, a distribution of monomer and dimer occurred and were similar to those when monomer undergoes hydrolysis to form the dimer. This supports the possibility that the dimer is stabilized with respect to the monomer in RMs.

**Figure 6:**
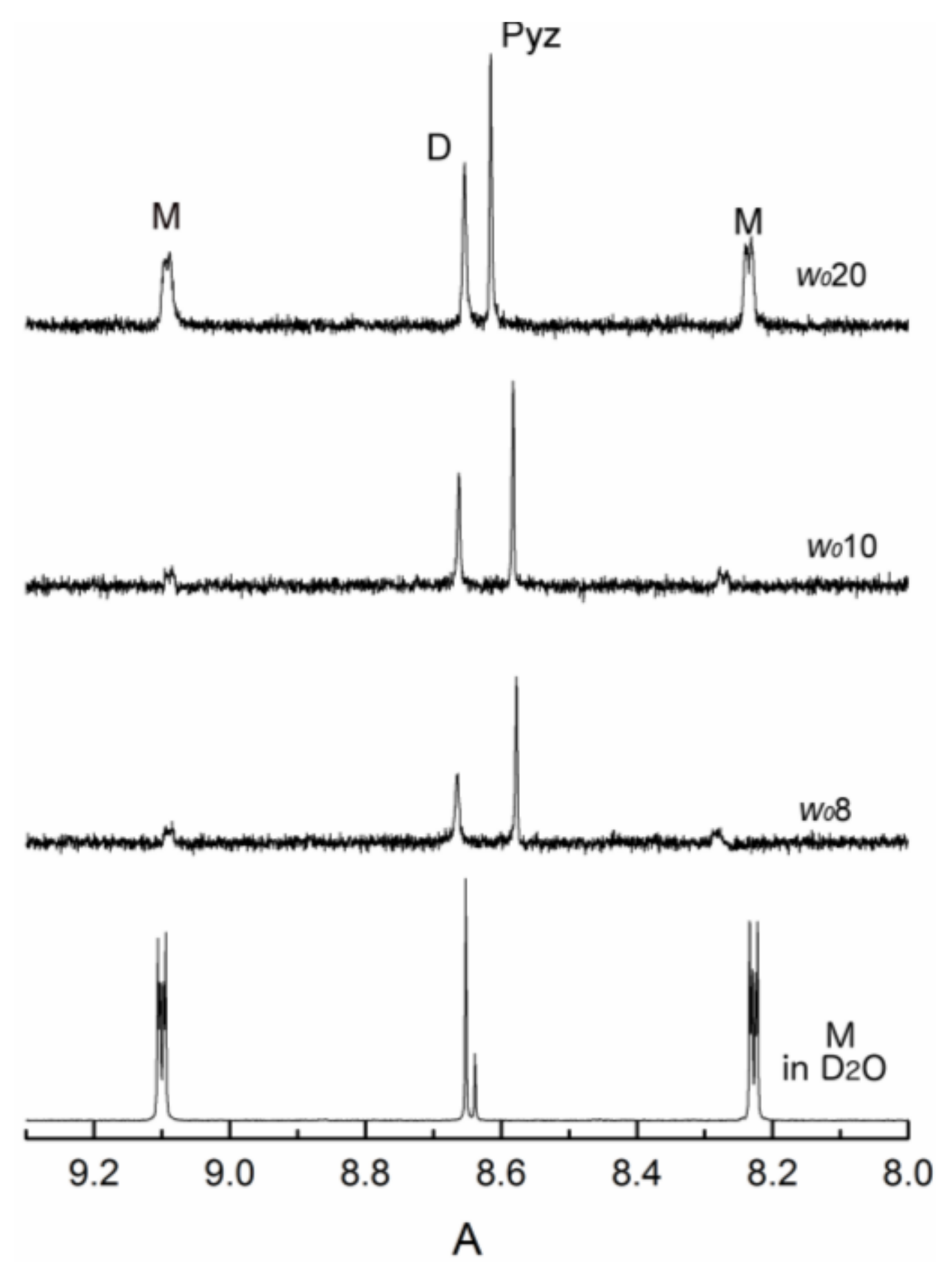
^1^H-NMR spectra recorded at 300 MHz of 0.020 M and in 0.75 M AOT/isooctane at pH 8.82. Starting from the bottom: M in D_2_O, M in RM sizes *w*_0_ 8, 10, and 20. Spectra were taken approximately 30 min after preparation of the mixture.

Experiments were carried out to ascertain the effect of RM size variation on the distribution of the monomer and dimer in the water pool. Figure 6 shows ^1^H-NMR spectra recorded for the monomer in varying *w*_0_ s (8, 10, and 20) with the position of the chemical shifts in D_2_O for comparison. In the RMs of *w*_0_ 8 and 10, the doublet signals for the monomer shift from those in D_2_O but not for *w*_0_ 20. Similarly, the dimer shifts at *w*_0_ 8 and 10 but not at *w*_0_ 20. This shows that the probes reside in the water pool and that the “aqueous” environment, e.g., the solvation, at *w*_0_ 20 is similar to that in bulk aqueous media. The shifts of ~0.06 ppm observed in the smaller *w*_0_ s can be rationalized by a change in the nature of water in aqueous core as the size is decreasing as well as the ion pairing due to the increased concentration of the sodium ions from the AOT surfactant. This ion pairing may result in more “rigidity” of the dimer structure via charge neutralization.

Our data are consistent with the monomer and the dimer residing in the water pool, although the possibility of interface proximity and some interaction at the interface cannot be completely discounted. Penetration of the complex or its pyrazine ligand into the negatively charged interfacial region however, seems unlikely, particularly for the high negatively charged dimer. Similar conclusions about changes in the core water, hence solvation, have been suggested for other AOT-iso-octane RM studies [26,46,47,48,49]. In contrast, the free pyrazine is shifted at all values measured as compared to bulk D_2_O. While not definitive, this suggests that pyrazine may reside closer to the interfacial regions between bulk water and the AOT head groups compared to the complexes. This difference between free and coordinated pyrazine is not unexpected since the metal complexes possess negative charges that will be electrostatically repelled by the negatively charged AOT head groups whereas the neutral pyrazine can move freely and associate favorable in the less polar interfacial region of the interface.

The ratio of monomer and dimer signals are dramatically altered upon confinement within the RM. The line broadening of the monomer and the dimer signals in the RMs suggest that the mobility of the complexes is significantly changed compared to the aqueous solution. The ^1^H-NMR spectra were recorded with different relaxation times (from 0.5 to 2 s) to prevent using inappropriate parameters when comparing the integration of the signals in different RM values to determine the ratios. Specifically, the integration ratios of the peaks in these spectra give a monomer:dimer ratio of 1.4 for D_2_O and 1.1, 0.13, 0.14 for *w*_0_ 20, 10, and 8, respectively. At small RM sizes, the monomer is almost completely converted into the dimer resulting in very small ratios so even if there are small change in the T_1_ values as the RM size decrease (data not shown), the observed pattern holds. 

**Figure 7:**
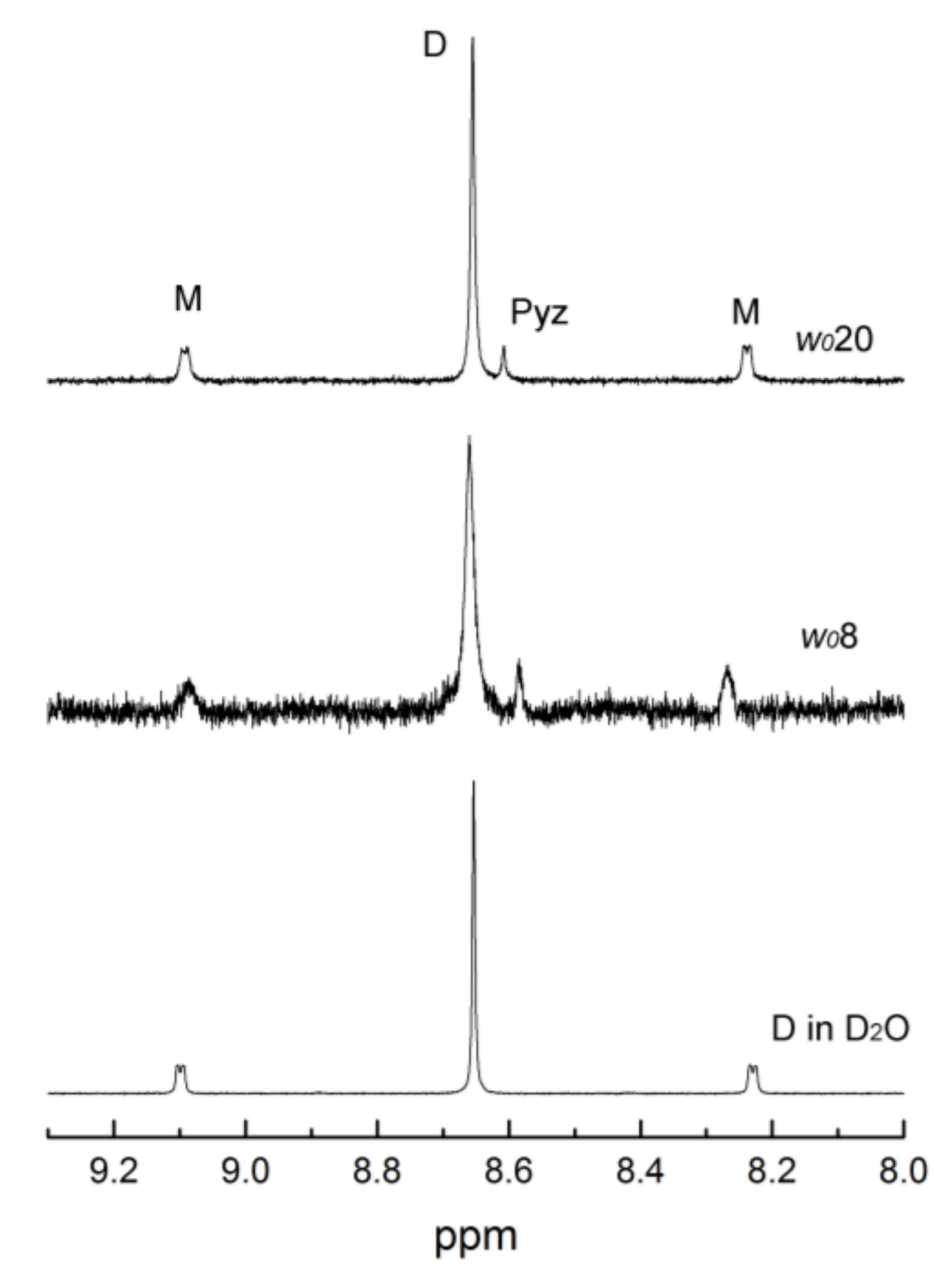
^1^H-NMR spectra were recorded at 300 MHz of a pH 9.0 solution D (0.020 mM) in aqueous solution and in 0.75 M AOT/isooctane RMs at *w*_0_ sizes 8, 10 and 20. All spectra were taken within 30 min of preparation.

Figure 7 shows the ^1^H-NMR of the dimer in D_2_O and in two AOT/isooctane RMs sixes. These spectra compare to a similar series of spectra previously shown in Figure 5. As expected, the aqueous solution shows the prominent signal for the dimer signal and minor signals for the monomer. The ratios of the monomer and dimer integrations equal 0.11 for both *w*_0_ 8 and 20. This ratio (0.13) is close to the ratio of the peaks measured when dissolving the monomer into solution. These two results support the UV-Vis studies in concluding that the dimer is stabilized over the monomer in RMs which is opposite that found in bulk water. In contrast to the spectra recorded from samples beginning with the monomer, the ratios of the dimer:monomer remain relatively constant even at *w*_0_ 8, where the monomer was unstable (see Figure 5 and Figure 6). However, the increase in linewidth on placement in the RM is consistent with a significant decrease in the relaxation time and a decreased tumbling rate in the RMs. Comparing the spectra of *w_0_* 8 and 20 show the increased linewith at the smaller RM and decreased tumbling in these smaller RMs. The appearance of a peak at 8.63 ppm corresponds to the formation of some free pyrazine that formed from the dissociation of the dimer. This supports our previous observations that the dimer is stabilized within a RM and only a little dimer is hydrolyzed when placed in the RM.

The significantly increased stability of the dimer in RMs, at the expense of the monomer, was not anticipated even though entropically, dimer would be favored over monomer. The decreased stability of the monomer with a 3-charge compared to the dimer with a 6-charge inside a RM with a negatively charged headgroup is however conceptually unexpected, and the observation could not be explained based on changes in concentration as the size of the RM changed. Using standard bond lengths, the diameters of the complexes range from 5–6 Å for the monomer to 13–15 Å for the dimer. The size of the RM waterpool have previously been reported [32] to have a diameter of 32 Å for the *w*_0_ 8 and 70 Å for the *w*_0_ 20. The size of the RM can be measured using Dynamic Light Scattering (DLS), and thus include both the waterpool and the interfacial layer of the surfactant. However, as the size approaches *w*_0_ 8, the dimer fills the entire RM with a shell of water molecules between the compound and the interface of the RM.

We have previously shown that V_10_ with a 6-charge is stable inside RMs and forms even when initially protonated [50]. We found that RMs could not be formed below *w*_0_ 5 or 6 in that system. This size allowed for a V_10_ molecule and a layer of 2–3 water molecules inside the water droplet. We also showed that a V_10_ with a Mo [51] replacing one of the V-atoms, V_9_Mo, with similar shape but at a different charge behave similarly to V_10_ [50,51]. These compounds are placed in the center of the water pool, however, we found that cations could change the system significantly [52]. Similarly, it was demonstrated that the interacting of water in NaAOT is compromised and that by comparison to the ionic liquid-like surfactant 1-butyl-3-methylimidazolium 1,4-bis-2-ethylhexylsulfosuccinate RMs in benzene that H-bonding has been disrupted in the NaAOT nanostructures [53]. Compounds such as aniline [29], phenol [50] or 1-phenylbiguanide [54] are found to associate closely with the interface, and thus it is possible that Pyr would similarly associate with the interface. Indeed, a recent molecular dynamic study has shown that there is strong interaction between solvent molecules at the interface region [55].

In conclusion, these types of studies are important for investigation of reactions in RMs. Specifically, protonation reactions [56,57,58,59], nucleophilic substitution reactions [60,61,62,63,64], electron transfer reactions [26,27,28,29], and enzymatic reactions [65,66,67] have been investigated and are known to be affected by the inhomogeneous environment such as those found in microemulsions.

## 3. Materials and Methods

### 3.1. General Materials and Methods

Chemicals were used as obtained or as in the case of sodium bis(ethylhexyl)sucinatte (also named dioctylsulfosuccinate sodium salt abbreviated NaAOT) purified according to previous methods [68]. All of the following chemicals were obtained from: NaAOT (Sigma-Aldrich (98% purity St. Louis, MO, USA), deuterium oxide (Cambridge Isotope Laboratories Inc., 99.9%, Tewksbury, MA, USA), 3-(trimethylsilyl)-1-propanesulfonic acid sodium salt (DSS) (Willard 99.9%, Frederick, MD, USA), 2,2,4-trimethylpentane (or isooctane) (Sigma-Aldrich ACS Reagent grade + 99.0%), methanol (EMD HPLC Grade 99.9%), pyrazine (Aldrich 99+% abbreviated pyz), d^6^-dimethylsulfoxide (Cambridge Isotope Laboratories Inc., 99.9% + 0.05% *v*/*v* TMS). The dimer, Na_6_[Fe_2_(CN)_10_pyz] and the monomer, Na_3_[Fe(CN)_5_pyz)] were synthesized and characterized as reported in the literature [44,45]. 

### 3.2. Preparation of Solutions

#### 3.2.1. Metal Complex Solutions

Aqueous solutions were prepared by dissolving the monomer or dimer into either 100% D_2_O or 10% D_2_O, unless otherwise indicated. The pH was adjusted using DCl or NaOD as needed. The pD values were measured with a pH meter and converted to pH by the relationship pH = pD + 0.40 [29].

#### 3.2.2. Reverse Micelle (RM) Solutions for Spectroscopic Measurements

Microemulsions of RMs were made using purified AOT and were prepared as a 0.75 M AOT/Isooctane solution. Subsequently, the appropriate amount of water (or aqueous probe solution) was added to form the desired RM size (*w*_0_ value = [H_2_O]/[AOT]) [32,69]. Residual amounts of water in the NaAOT preparation was accounted for in the reported *w*_0_ values and were generally around 0.25–0.30 water molecules for each AOT molecule. UV-Vis measurements were made using similarly prepared RMs, but the total AOT concentration was 0.20 M. The monomer or dimer was added to the RM using a water (or D_2_O)/complex solution in place of the pure water (or D_2_O). The resulting mixture was then vortexed until the solution was transparent. These samples were then immediately subjected to investigation by NMR spectroscopic methods.

### 3.3. Methods

#### 3.3.1. UV-Visible Spectroscopy

Solutions for analysis was prepared as described above. The concentrations of the metal complex ranged between 5–10 × 10^−5^ M. The UV-Vis spectra were collected using an HP8452A spectrometer (Waltham, MA, USA). Spectra were recorded within 1 min after mixing and at various times thereafter. The spectrometer was referenced using solutions of “empty” RMs at the same *w*_0_ size as those being studied containing the probe. This blank was found to give better baseline readings than recording against air. The use of a “blank” is necessary since there is some light scattering due to the RMs in solution [39].

#### 3.3.2. ^1^H-NMR Spectroscopy

^1^H-NMR spectra were recorded using a 300 or 400 MHz NMR Varian spectrometer (Palo Alto, CA, USA). Spectra for the monomer and dimer RM solutions were replicated three times. Samples were made fresh each time and immediately shielded from light. Spectra were recorded within 10 min of mixing unless otherwise indicated; significant changes in samples were observed over 30 min to 1 h. The parameters use for recording these spectra in RMs had sweep widths of 5200 Hz, relaxation delays of 1.0 s and a 45–60° pulse angle. The samples were locked on D_2_O (100%) and referenced against an AOT/isooctane signal which had previously been referenced against a solution of DSS as described previously [27].

#### 3.3.3. Dynamic Light Scattering (DLS)

DLS measurements were done to confirm that RMs formed and that they were of the sizes reported previously [32].

## 4. Conclusions

In this study, we have demonstrated that a confined environment affects the stability of simple coordination complexes, i.e., their ligand exchange equilibria, along with their speciation. We observed that the complexation rates are increased as the size of the RM decreases and that the dimeric form of the complex is the preferred species in the reverse micellar environment in contrast to bulk water where the monomer is the dominant species.

The changes observed in the NMR spectra, and differences in equilibria, for the different complex forms added to a variety of RM sizes could be due to environmental factors within the RMs. Specifically, the solvation of the metal complexes may control their hydrolysis rates and speciation as the size of the RM changes. At low *w*_0_ values, there are fewer water molecules and these are used primarily to solvate the sodium ions and AOT headgroups [32], thus forming the RM. Introduction of a metal complex will compete for these waters and change its dynamics. The shifting NMR peaks observed between water and in different RMs is consistent with this conclusion, although the observed changes may also be in part due, in part, to increased ion pairing with the sodium ions or interactions with the RM headgroups. Nevertheless, it is clear that the environment in which the metal complex is confined differs significantly from bulk water. 

This study imparts insight into how metal complexes behave at interfaces and as solvation is altered at the nanoscale. These insights will be important in the understanding of how molecular species are affected and interact at interfaces or membranes within biological, geological, or industrial systems [70,71,72,73,74,75,76].
